# Quantitative analysis of choroidal vasculature in central serous chorioretinopathy using ultra-widefield swept-source optical coherence tomography angiography

**DOI:** 10.1038/s41598-022-23389-1

**Published:** 2022-11-01

**Authors:** Qiaozhu Zeng, Yuou Yao, Shu Tu, Mingwei Zhao

**Affiliations:** 1 Department of Ophthalmology, Eye Diseases and Optometry Institute, Peking University People’s Hospital, Beijing, China; 2 Beijing Key Laboratory of Diagnosis and Therapy of Retinal and Choroid Diseases, College of Optometry, Peking University Health Science Center, Beijing, China

**Keywords:** Retinal diseases, Diseases

## Abstract

We aimed to quantitatively compare the choroid blood flow and choroid thickness at the periphery among eyes with central serous chorioretinopathy (CSC), fellow eyes and healthy eyes using ultra-widefield swept-source optical coherence tomography angiography (UWF SS-OCTA). Retrospective analysis of 49 patients with CSC (98 eyes, including unaffected fellow eyes) and 49 age and sex matched controls were included. We obtained 3-dimensional data of vertical 20 mm × horizontal 24 mm × scan depth 6 mm, comprising 9 subfields (superotemporal, upper, superonasal, temporal, central, nasal, inferotemporal, lower, inferonasal regions). CSC eyes presented with greater density of large-vessel choroidal layer in all the 9 subfields compared with controls. Compared with normal eyes, CSC eyes had greater choroidal thickness (superotemporal, upper, superonasal, temporal, central, nasal, inferotemporal, and inferonasal subfields) and choroidal volume (superotemporal, upper, superonasal, temporal, central, and nasal subfields). Compared with control eyes, the choriocapillaris density in the superotemporal, inferotemporal and inferonasal subfields was greater in patients with CSC. Our study may provide further evidence for the congestion of vortex vein in the pathogenesis of CSC. UWF SS-OCTA can be used to evaluate the abnormalities of the choroidal structures even at the periphery in eyes with CSC.

## Introduction

Central serous chorioretinopathy (CSC) is a representative pachychoroid spectrum disease characterized by serous retinal detachment with or without retinal pigment epithelium (RPE) detachment, which often occurs in young to middle-aged persons^1,2^. The pathogenesis of CSC has not yet been clearly elucidated. Retinal pigment epithelium (RPE) dysfunction was considered to be the main cause of CSC, with sites of leakage at the level of RPE in the fluorescein angiograms (FA)^3^. However, choroidal vascular abnormalities are proposed as the primary cause of alterations of CSC at present, revealed by choriocapillaris congestion, increased choroidal permeability, leakage into interstitial or stromal space, and dilatation of choroidal vessels in the indocyanine green angiograms^4–7^. Recently, several reports have suggested vortex vein congestion to play a role in CSC, including an asymmetric pattern of superior and inferior vortex veins^8–10^, anastomosis between superior and inferior vortex veins and etc.

Advancements of ultra-widefield swept-source optical coherence tomography angiography (UWF SS-OCTA) enables clinicians to observe the choroid in more peripheral areas of the fundus. An UWF SS-OCTA device is available from TowardPi Medical Technology (TowardPi Medical Technology Co., Ltd, Beijing, China): BM400K BMizar. With the combination of long wavelength (1060 nm) full range swept source and 400 kHz A-scan rate, the device has capability to acquire as deep as 6 mm scan depth, scan range of 24 mm × 20 mm and the largest field of view of 81° × 68°. It involves the vicinity of the vortex vein ampulla which may promote the exploration of peripheral choroidal structures in eyes with CSC. It could provide additional information on the choroid supply in CSC, and therefore help us better understand the pathophysiology of the disease.

In order to obtain the characteristic findings suggesting the pathogenesis of CSC, we aim to quantitatively compare the choroid blood flow and choroid thickness at the periphery among eyes with CSC, fellow eyes and healthy eyes using the images obtained by UWF SS-OCTA.

## Results

This study included 49 patients (36 men and 13 women) with unilateral CSC. The mean duration of symptoms was 3.4 ± 3.5 months. No obvious OCT(A), FA, and fundus auto-fluorescense (FAF) findings suggestive of CSC were revealed in 49 fellow eyes. There were 34 and 15 patients with acute and chronic CSC, respectively. Forty-nine eyes of 49 age and sex matched healthy subjects (36 men and 13 women) were included as controls. Demographics of the participants were presented in Table 1. Age, sex, and proportions of hypertension and diabetes were insignificantly different between the patients with CSC and controls. Comparisons of characteristics among CSC eyes, fellow eyes and healthy control eyes were shown in Table 2. The IOP, AL and spherical equivalent did not differ among the three groups. Compared with the BCVA of normal eyes, those of patients with CSC were significantly worse (P = 0.001 for fellow eyes; P < 0.001 for eyes with CSC). The BCVA was also statistically worse in the diseased eyes of CSC than fellow eyes (P < 0.001).

**Table 1:** Characteristics of patients with CSC and healthy subjects.

Parameters	Patients with CSC	Healthy subjects	P-value
No. (male/female)	49 (36/13)	49 (36/13)	> 0.05
No. of eyes	49	49	NA
Age, years, mean ± SD	45.5 ± 9.3	43.5 ± 12.9	0.388
Hypertension, n (%)	6 (12.2)	4 (8.2)	0.505
Diabetes, n (%)	1 (2.0)	4 (8.2)	0.168
Duration of symptoms, months, mean ± SD	3.9 ± 3.6	NA	NA

*CSC* central serous chorioretinopathy, *NA* not applicable, *SD* standard deviation.

**Table 2:** Comparisons of parameters among CSC eyes, fellow eyes and healthy control eyes.

	CSC eyes	Fellow eyes	Healthy control eyes	P-value^1^	P-value^2^	P-value^3^
Best corrected visual acuity in logMAR, IQR, range	0.1 (0,0.3)	0 (0,0.5)	0 (0,0)	0.001*	< 0.001*	< 0.001*
Intraocular pressure, mmHg, mean ± SD	14.6 ± 2.7	14.7 ± 2.6	16.3 ± 3.9	0.418	0.070	0.088
Axial length, mm, mean ± SD	23.8 ± 1.0	23.9 + 1.1	24.2 ± 1.1	0.218	0.164	0.309
Spherical equivalent, diopters, mean ± SD	− 0.7 ± 2.0	− 0.7 ± 2.1	− 0.9 ± 1.7	0.993	0.540	0.584

*CSC* central serous chorioretinopathy, *logMAR* logarithm of the minimum angle of resolution, *SD* standard deviation.

*Statistically significant.

^1^Comparisons of ocular factors between diseased and fellow eyes of patients with CSC were performed using the paired t test for parameters with normal distribution and using the Wilcoxon matched-pairs signed rank test for parameters with nonnormal distribution.

^2^Comparisons of ocular factors between healthy eyes and fellow eyes of patients with CSC were performed using the independent t test for parameters with normal distribution and using the Mann Whitney U test for parameters with nonnormal distribution.

^3^Comparisons of ocular factors between healthy eyes and diseased eyes of patients with CSC were performed using the unpaired t test for parameters with normal distribution and using the Mann Whitney U test for parameters with nonnormal distribution.

**Figure 1:**
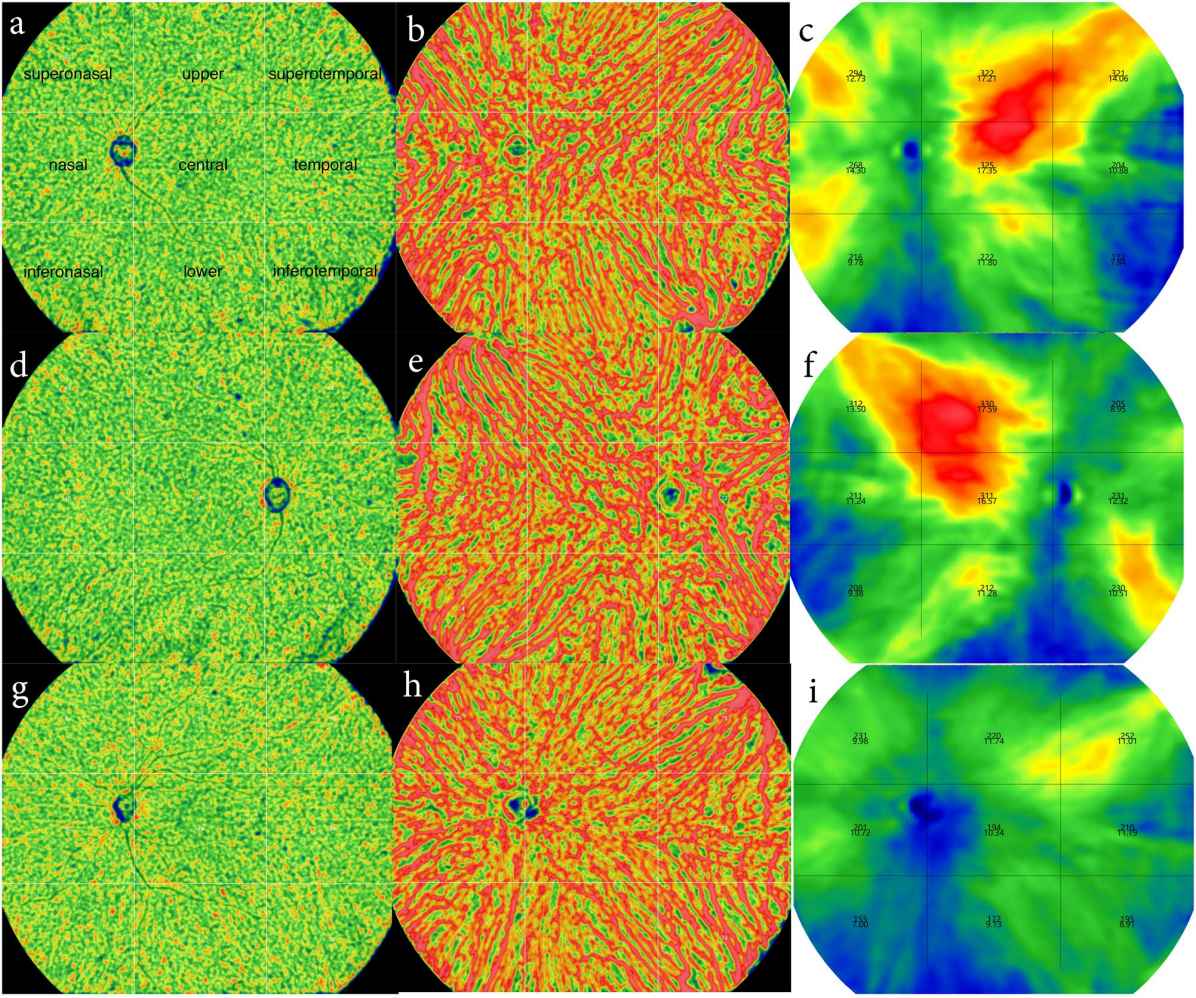
Representative maps of choriocapillaris density (**a**,**d**,**g**), large-vessel choroidal layer density (**b**,**e**,**h**), choroidal thickness, choroidal vessel volume (**c**,**f**,**i**) in CSC (**a**–**c**), fellow (**d**–**f**) and control (**g**–**i**) eyes.

The UWF SS-OCTA provided maps of choriocapillaris density, large-vessel choroidal layer density, choroidal thickness, choroidal vessel volume involving the vicinities of the vortex vein ampullae in both patients with CSC and controls (Fig. 1).

Table 3 summarizes the comparisons of large-vessel choroidal layer density, choroidal thickness, choroidal vessel volume and choriocapillaris density among the CSC, fellow and control eyes. CSC eyes presented with greater density of large-vessel choroidal layer in all the subfields (superotemporal [72.3 ± 2.9% vs 66.5 ± 5.2%, P < 0.001], upper [71.3 ± 2.0% vs 70.0 ± 2.5%, P = 0.004], superonasal [71.2 ± 3.2% vs 65.2 ± 6.8%, P < 0.001], temporal [70.9 ± 2.2% vs 69.4 ± 1.8%, P < 0.001], central [72.0 ± 2.7% vs 70.1 ± 2.5%, P < 0.001], nasal [70.3 ± 2.9% vs 68.7 ± 3.4%, P = 0.004], inferotemporal [72.0 ± 3.2% vs 66.5 ± 5.9%, P < 0.001, lower [69.7 ± 4.6% vs 68.2 ± 3.7%, P = 0.002] and inferonasal [69.6 ± 4.7% vs 62.3 ± 6.8%, P < 0.001]), compared with controls. Density of large-vessel choroidal layer in CSC eyes was also greater in the upper (71.3 ± 2.0% vs 70.7 ± 2.3%, P = 0.002), central (72.0 ± 2.7% vs 71.3 ± 3.2%, P = 0.018), and nasal (70.3 ± 2.9% vs 69.4 ± 3.5%, P = 0.002) subfields than that in fellow eyes. Compared with the choroidal thickness of normal eyes, those of eyes with CSC were significantly greater in superotemporal (279.2 ± 65.0 µm vs 222.7 ± 41.0 µm, P < 0.001), upper (258.7 ± 61.8 µm vs 219.1 ± 47.7 µm, P = 0.001), superonasal (230.5 ± 59.6 µm vs 195.9 ± 55.7 µm, P = 0.004), temporal (313.9 ± 88.6 µm vs 256.8 ± 57.5 µm, P = 0.009), central (350.4 ± 107.3 µm vs 277.4 ± 78.8 µm, P < 0.001), nasal (229.3 ± 63.5 µm vs 277.4 ± 78.8 µm, P = 0.001), inferotemporal (266.1 ± 91.7 µm vs 229.9 ± 56.1 µm, P = 0.037) and inferonasal (166.8 ± 44.3 µm vs 148.4 ± 35.9 µm, P = 0.027) subfields of the measurement grid. The choroidal thicknesses of eyes with CSC, with the exception of the inferotemporal and superonasal subfields, were significantly greater than those of fellow eyes. Compared with the choroidal volume of normal eyes, those of eyes with CSC were significantly greater in superotemporal, upper, superonasal, temporal, central, nasal subfields. The choroidal volume of superotemporal, upper, temporal, central, nasal, lower and inferonasal subfields was greater in CSC eyes than in fellow eyes. Compared with control eyes, the density of choriocapillaris in the supertemporal (47.1 ± 1.9% vs 45.7 ± 3.1%, P = 0.006), inferotemporal (48.1 ± 1.5% vs 45.5 ± 4.0%, P = 0.007) and inferonasal (47.2 ± 1.8% vs 45.2 ± 3.1%, P = 0.002) subfields respectively was greater in eyes with CSC. Table 4 indicated that the choriocapillaris density in central subfield was similar to that in extra-central subfield in normal eyes (45.6 ± 1.1% vs 46.2 ± 1.8%, P = 0.057), while in the CSC (46.0 ± 1.1% vs 47.0 ± 1.1%, P < 0.001) and fellow eyes (45.7 ± 1.1% vs 46.8 ± 1.3%, P < 0.001), the choriocapillaris density in central subfield was less than that in extra-central subfield. There was positive correlation between choroidal thickness and choroidal volume in all the participants with correlation coefficients of more than 0.8 (P < 0.001). Choriocapillaris vessel density showed no correlations with large-choroidal vessel density in the three groups.

**Table 3:** Comparisons of choroidal vasculature in nine regions among CSC eyes, fellow eyes and healthy control eyes.

	CSC eyes	Fellow eyes	Control eyes	P-value^1^	P-value^2^	P-value^3^
**Large choroid vessel density, %, mean ± SD**						
Superotemporal	72.3 ± 2.9	71.8 ± 2.6	66.5 ± 5.2	0.061	< 0.001*	< 0.001*
Temporal	71.3 ± 2.0	70.7 ± 2.3	70.0 ± 2.5	0.002*	0.094	0.004*
Inferotemporal	71.2 ± 3.2	70.7 ± 3.2	65.2 ± 6.8	0.257	< 0.001*	< 0.001*
Upper	70.9 ± 2.2	70.6 ± 2.1	69.4 ± 1.8	0.125	0.004*	< 0.001*
Central	72.0 ± 2.7	71.3 ± 3.2	70.1 ± 2.5	0.018*	0.005*	< 0.001*
Lower	70.3 ± 2.9	69.4 ± 3.5	68.7 ± 3.4	0.002*	0.103	0.004*
Superonasal	72.0 ± 3.2	71.8 ± 2.7	66.5 ± 5.9	0.186	< 0.001*	< 0.001*
Nasal	69.7 ± 4.6	69.0 ± 5.4	68.2 ± 3.7	0.146	0.028*	0.002*
Inferonasal	69.6 ± 4.7	68.1 ± 5.9	62.3 ± 6.8	0.067	< 0.001*	< 0.001*
Average	71.0 ± 2.7	70.4 ± 2.9	67.4 ± 3.4	0.005*	0.1	< 0.001*
**Choroidal thickness, μm, mean ± SD**						
Superotemporal	279.2 ± 65.0	260.9 ± 62.9	222.7 ± 41.0	0.013*	0.001*	< 0.001*
Temporal	258.7 ± 61.8	237.8 ± 54.5	219.1 ± 47.7	< 0.001*	0.074	0.001*
Inferotemporal	230.5 ± 59.6	215.6 ± 53.6	195.9 ± 55.7	0.095	0.052	0.004*
Upper	313.9 ± 88.6	290.6 ± 79.9	256.8 ± 57.5	0.006*	0.046*	< 0.001*
Central	350.4 ± 107.3	312.0 ± 107.0	277.4 ± 78.8	< 0.001*	0.071	< 0.001*
Lower	229.3 ± 63.5	211.3 ± 57.8	190.1 ± 51.5	< 0.001*	0.058	0.001*
Superonasal	266.1 ± 91.7	249.9 ± 72.3	229.9 ± 56.1	0.087	0.129	0.037*
Nasal	239.7 ± 86.8	220.3 ± 76.4	218.2 ± 59.8	0.007*	0.882	0.157
Inferonasal	166.8 ± 44.3	156.7 ± 42.5	148.4 ± 35.9	0.016*	0.301	0.027*
**Choroidal volume, mm**^**3**^**, mean ± SD**						
Superotemporal	12.2 ± 2.8	11.4 ± 2.8	10.4 ± 2.0	0.018*	0.1	0.003*
Temporal	13.8 ± 3.3	12.8 ± 3.1	11.7 ± 2.5	< 0.001*	0.054	0.001*
Inferotemporal	10.5 ± 2.7	9.8 ± 2.5	9.4 ± 2.6	0.118	0.375	0.0495*
Upper	16.8 ± 4.7	15.5 ± 4.3	13.7 ± 3.1	0.005*	0.046*	< 0.001*
Central	18.7 ± 5.7	16.7 ± 5.7	14.9 ± 4.1	< 0.001*	0.087	< 0.001*
Lower	12.2 ± 3.4	11.3 ± 3.1	10.1 ± 2.7	< 0.001*	0.057	0.001*
Superonasal	11.6 ± 4	10.9 ± 3.2	10.9 ± 2.7	0.14	0.903	0.555
Nasal	12.8 ± 4.6	11.8 ± 4.1	11.7 ± 3.2	0.006*	0.885	0.157
Inferonasal	7.6 ± 2.0	7.2 ± 1.9	7.1 ± 1.8	0.035*	0.957	0.317
**Choriocapillaris density, %, mean ± SD**						
Superotemporal	47.1 ± 1.9	46.9 ± 2.1	45.7 ± 3.1	0.171	0.096	0.006*
Temporal	45.9 ± 1.5	45.7 ± 1.5	46.1 ± 1.3	0.257	0.125	0.471
Inferotemporal	46.1 ± 1.9	46.1 ± 2.1	45.1 ± 3.9	0.838	0.102	0.076
Upper	47.2 ± 1.2	47.1 ± 1.6	47.5 ± 1.2	0.849	0.113	0.148
Central	46.0 ± 1.1	45.7 ± 1.1	45.7 ± 1.1	0.065	0.994	0.284
Lower	47.3 ± 1.5	47.2 ± 1.5	47.6 ± 1.1	0.441	0.052	0.121
Superonasal	48.1 ± 1.5	48 ± 1.9	45.5 ± 4.0	0.912	0.003*	0.007*
Nasal	47 ± 1.2	47 ± 1.1	47.2 ± 1.1	0.907	0.325	0.395
Inferonasal	47.2 ± 1.8	46.9 ± 2.4	45.2 ± 3.1	0.679	0.015*	0.002*
Average	46.9 ± 1.1	46.7 ± 1.2	46.2 ± 1.6	0.228	0.1	0.025*

Data are presented as means ± standard deviations unless otherwise indicated.

^1^Comparisons of ocular factors between diseased and fellow eyes of patients with CSC were performed using the paired t test for parameters with normal distribution and using the Wilcoxon matched-pairs signed rank test for parameters with nonnormal distribution.

^2^Comparisons of ocular factors between healthy eyes and fellow eyes of patients with CSC were performed using the independent t test for parameters with normal distribution and using the ManneWhitney U test for parameters with nonnormal distribution.

^3^Comparisons of ocular factors between healthy eyes and diseased eyes of patients with CSC were performed using the unpaired t test for parameters with normal distribution and using the ManneWhitney U test for parameters with nonnormal distribution.

**Table 4:** Comparisons of choriocapillaris density between central and extra-central subfields in CSC, fellow and control eyes.

Variable	Groups	Extra-central	Central	P-value
Choriocapillaris density, %	CSC, mean ± SD	47.0 ± 1.1	46.0 ± 1.1	< 0.001^2^
	Fellow, mean ± SD	46.8 ± 1.3	45.7 ± 1.1	< 0.001^2^
	Control, mean ± SD	46.2 ± 1.8	45.6 ± 1.1	0.057^2^

Data are presented as means ± standard deviations unless otherwise indicated.

*CSC* central serous chorioretinopathy.

^1^The paired t test for parameters with normal distribution.

^2^The Wilcoxon matched-pairs signed rank test for parameters with nonnormal distribution.

Demographics of the acute CSC (aCSC) and chronic CSC (cCSC) patients were presented in Supplementary Table S1. cCSC presented with a significantly older year of age (43.3 ± 7.5 vs 50.3 ± 11.3, P = 0.014). Sex, and proportions of hypertension and diabetes were not different between the acute and chronic CSC. Comparisons of characteristics between acute and chronic CSC eyes were indicated in Supplementary Table S2. The IOP, AL and spherical equivalent did not differ between the two groups. However, the BCVA of patients with cCSC were statistically worse (0.13 ± 0.15 vs 0.47 ± 0.53, P = 0.003). As shown in Supplementary Table S3, aCSC eyes presented with greater density of large-vessel choroidal layer in the upper (71.3 ± 1.9% vs 69.9 ± 2.5%, P = 0.034), superonasal (72.9 ± 1.8% vs 69.9 ± 4.5%, P = 0.002), temporal (71.9 ± 1.5% vs 70.1 ± 2.5%, P = 0.004), central (72.6 ± 2.0% vs 70.7 ± 3.4%, P = 0.017) and inferotemporal (72.0 ± 2.5% vs 69.3 ± 4.0%, P = 0.006) subfields. Compared with the choroidal thickness and volume of chronic eyes, those of eyes with aCSC were significantly greater in upper, superonasal and temporal regions. The choriocapillaris density of most subfields were indifferent between chronic and acute CSC, with the exception of inferotemporal subfield (acute vs chronic: 45.7 ± 1.9 vs 47 ± 1.7, P = 0.03). In both the acute and chronic CSC eyes, the choriocapillaris density in central subfield was less than that in extra-central subfield, as shown in Supplementary Table S4.

## Discussion

We quantitatively compared the choroidal thickness, choroidal volume, large-choroidal vessel density and choriocapillaris density among CSC eyes, fellow eyes and control eyes using UWF SS-OCTA.

There have been few studies evaluating the ultra-widefield choroidal morphology in CSC patients. With the UWF-OCT, Izumi et al. proposed that the subfoveal choroid in CSC eyes was significantly thicker than that in normal eyes, while no difference at the periphery was found^11^. In the study by Ishikura et al., they found that, compared with that in normal eyes, the choroidal thickness in eyes of patients with CSC was statistically greater in all subfields^12^. The choroidal thicknesses in eyes of patients with CSC were greater than that in fellow eyes, except for the outer superotemporal and inferonasal subfields.

In our study, it was detected that the choroid thickness at the periphery and posterior pole was greater in CSC eyes than fellow or control eyes in all the subfields except for the lower field. This difference with aforementioned studies may be attributed to the fact that their evaluation was on the basis of different methodology, including OCT-B-scans, or subfields of a grid consisting of inner and outer rings. UWF SS-OCTA applied in our study helped us measure the choroidal thickness involving the vicinity of the vortex vein ampulla, which was always located in the superonasal, superotemporal, inferonasal and inferotemporal regions. Similarly, we revealed that large-choroidal vessel density was higher in all the subfields in CSC eyes than in control eyes, especially the superotemporal, superonasal, inferotemporal and inferonasal subfields with P-value less than 0.001. Previous study proposed that the anastomoses in CSC occurred nearly uniformly among the superonasal, superotemporal, and inferotemporal vortex vein systems^10,13,14^. The relatively asymmetric distribution of choroidal thickness, choroidal volumes and large-choroidal vessel density found in our study may further support the view that the pathogenesis of typical CSC could involve congestion due to the impaired drainage of the affected vortex veins^1,8,10,14–16^, consistent with the result of Ishikura et al.’s study^12^. In the study by Nishihara et al.^17^, they found the choroidal thickness was significantly thicker in the eyes with CSC than that in the normal eyes at subfoveal choroidal thickness, at 5 mm superior, and at 7 mm superior from the fovea. However, there was no significant difference in the choroidal thickness at the more peripheral points including I5, I7, S8, I8, S10 and I10. Therefore, it was proposed that the choroid was not entirely thickened in eyes with CSC, and the choroid is thickened in the SCT and not at the periphery. Their results were not completely in accordance with those of our study, although we also found greater choroidal thickness in some superior fields. There could be several explanations for the difference in results between our study and the study by Nishihara et al. First, our study applied the three-dimensional choroidal thickness with wider fields to assess the peripheral OCTA in CSC, while Nishihara et al. used the vertical scans (20 mm) through the fovea. Therefore, the discrepancy in results could be easy-to-understand due to our different methods. Second, the three-dimensional measurement was more all-round but less specific than B-scan. We could carry out further study about the choroidal thickness in some specific points to evaluate the periphery of CSC. It could be possible that the choroid in some particular regions is not thickened in CSC. Third, the heterogeneity of sample size, duration of symptom, leakage point, region of choroidal vascular hyperpermeability and etc. could not be neglected. As stated in the study by Nishihara et al., it is not known whether CSC is caused by an abnormality in some specific choroidal tissue or whether it is an abnormality that can occur at all choroidal areas.

In contrary to previous studies^18–21^, we found that choriocapillaris density in the superotemporal, inferotemporal and inferonasal subfields was greater in CSC eyes, compared with that in control eyes, which was consistent with the published study by Kuroda et al.^22^. There could be several interpretations. On the one hand, the heterogeneity of sample size, duration of symptom, segmentations of choroid layers and algorithm could not be neglected. On the other hand, most of the previous examinations only assessed the macular area, whereas pathologic changes at the periphery have not been sufficiently investigated. Previous studies proposed that choriocapillaris flow deficits could originate from mechanical stress induced by compression caused by enlarged underlying choroidal vessels^23^, however, we found similar choriocapillaris density in the central subfield in CSC, fellow and control eyes. In addition, we did not find a negative correlation between choriocapillaris density and large-choroid vessel density in all the participants.

Spaide and et al. proposed the term “venous overload choroidopathy”, centered on venous overload and physiologic consequences induced in the choroid^20^. In CSC eyes, to ameliorate the congestion, the remodeling and anastomosis between the superior and inferior vortex veins more frequently developed. In addition, they innovatively proposed Starling resistor for choroidal vascular flow. The choroid can keep the choriocapillaris and larger veins filled with blood over a large range of intraocular pressure^24^. Increased hydrostatic pressure in choroids triggered the Starling resistor that narrowed the venous outflow near the vortex vein, contributing to creating a pathologic state such as choriocapillaris flow reduction by blocking venous outflow. The significant choriocapillaris flow void reduction only in the fovea lesion in CSC eye might demonstrate that a fovea is the region most sensitive and vulnerable to excessive choroidal blood inflow, leading to the reversible choriocapillaris flow deficit earlier than other areas. Similar to the Spaide’s theory, we revealed that large-choroidal vessel density was higher in all the subfields in CSC eyes than in control eyes, especially the superotemporal, superonasal, inferotemporal and inferonasal subfields. We speculate that vortex vein congestion may lead to anastomosis between the superior and inferior vortex veins. Remodeling of choroidal drainage routes by venous anastomosis between superior and inferior vortex veins may be common in CSC. Our study was a supplement to testify the theory of Spaide, which was based on quantitative measurements of ultra-widefield OCTA. In addition, we replenished the role of choriocapillaris layer in CSC, connecting with the aforementioned theory of Spaide. We innovatively confirmed the imbalance of choriocapillaris ischemia by comparing the central and extra-central choriocapillaris density in CSC, fellow and control eyes. In normal eyes, the choriocapillaris density in the fovea is theoretically maximal, rather than the whole choroid^25^, as opposed to the CSC and fellow eyes. In the periphery, the choriocapillaris is associated with only one deep layer of large blood vessels^26,27^. We assumed that the relatively higher choriocapillaris density in CSC and fellow eyes in our study may be attributed to the higher whole choroid blood density resulted from congestion of vortex veins.

Spaide and et al. proposed that there is more generalized outflow problem of the entire outflow system. Pang et al. revealed that dilatation of the ampulla in the dominant vortex vein suggests outflow disturbance through the scleral tunnel, which might be narrowed by the thickened sclera^28^. Recent studies reported the anterior scleral thickness was greater in eyes with CSC than in controls^29^. In CSC, the excess fluid permeates through the sclera to leave the eye. Increasing thickness would be expected to decrease the amount of fluid permeating the sclera. In addition, a thicker sclera would increase the length of vein compressed, potentially augmenting the Starling resistor. To better confirm our results and the connection with Spaide’s theory, using the advanced widefield OCT and OCTA, we could focus more on the status of choriocapillaris layer and scleral thickness in the future.

The chronic CSC is recognized by widespread significant multifocal areas of atrophic RPE alterations (including flat irregular PED, hyperreflective vascular walls, intrachoroidal dots, the double-layer sign, intraretinal cystoid cavities, choroidal neovascularization and etc.) and/or multifocal areas of leakage, leading to permanent visual impairment^30^. With prolonged duration and improper treatment, aCSC may progress into cCSC with diffuse RPE disease^1^. cCSC tends to have older age and female gender^1,30^. Consistently in our study, chronic cases were found to be significantly older than aCSC but both of the groups had male predominance. In addition, cCSC patients had worse BCVA than aCSC, due to the widespread RPE alterations. Similar to previous studies, we found higher density of choriocapillaris in cCSC than in aCSC at the inferotemporal subfield. We hypothesized that the hypoperfusion with surrounding hyperperfusion on OCTA may be the compensatory status due to ischemia in cCSC^31–33^. However, in contrary to some published studies^34,35^, we found the choroidal thickness and volume, as well as large-vessel density were higher in the aCSC group. We speculated that the choroidal large -vessel may be extensively dilated in the acute phase, and with the disease progressing, the swelling of the choroidal vessel and matrix may be alleviated. Congestion of vortex veins to possibly undergo gradual amelioration corresponding to anastomosis developing between the superior and inferior vortex veins as pachychoroid spectrum diseases progress^36^. Moreover, the different duration and phase in CSC patients in those studies could contribute to the heterogeneity of results. We may carry out longitudinal cohort with large sample size to evaluate the choroidal differences between acute and chronic CSC in the future.

SS-OCTA provides high-resolution and depth-resolved visualization of choroidal vasculature. However, the artifacts could not be neglected. Previous studies have defined imaging artifacts of SS-OCTA, such as segmentation, banding, motion, projection, masking, unmasking, doubling of the retinal vessels, blink, stretching, out-of-window and crisscross^37,38^. In our study, the UWF-SS-OCTA applied the novel “projection-resolved” (PR) algorithm”, which effectively suppresses the projection artifact on both en face and cross-sectional angiograms and enhanced depth resolution of vascular networks^39^. Therefore, the density of choriocapillaris layer will not be significantly influenced. In addition, we used an effective visualization technique (higher-order moments amplitude decorrelation angiography, HMADA) of both larger blood vessels and the capillary network in the choroidal circulations, by capturing higher order statistical signals in OCTA data. With artificial intelligence technology, each layer including BM and CSI is able to be recognized. Hence, the large choroid vessel density and volume or thickness of choroid will not be affected by projection. Moreover, motion artifacts could be derived from ocular pulsation, drift, blink and etc. To minimize the impact of motion, our UWF-SS-OCTA adopted the eye-tracking technique with 128 Hz, which is higher than most of the commercial OCTA with 15–30 Hz^40,41^. Signal attenuation could be derived from the more anterior dense lesions such as vitreous opacities, hemorrhage, scar tissue, pigment clumps, eye lashes and so on blocking the light to reach the deeper layers. Although modern deep-learning-based algorithms have demonstrated a capability to distinguish shadowing artifacts from real pathology, we excluded patients with any circumstances that may affect the quality of imaging (quality of OCT or OCTA < 7) such as cataract, hemorrhage, and etc. In addition, we lifted the eyelids of some patients to avoid effect of eye lashes. With the aforementioned advanced technology and strict inclusion criteria, we have minimized the influence of artifacts on measurement.

However, there are also several limitations in our study. First of all, the duration of symptoms from the onset was provided by patients, which may be not precise due to recall bias. Some acute CSCs may progress into chronic phase and recurrence within 1 year. Larger longitudinal study with acute, chronic, recurrent, or resolved CSC could be conducted in the future. Second, the distortion of UWF SS-OCTA in the periphery was inevitable and independent of segmentation, which may lead to the overestimation of choroidal parameters. The en-face images produced by the distorted 3D volumetric data may lead to 2D images with more distortion around the border. In our ultra-widefield SS-OCTA, the scanning mode was improved to reduce the distortion effect. To minimize the bias, we unified the measurement range among participants by correcting the AL-related magnification. In addition, the density was proportional parameter, which would be not impacted by the distortion. Moreover, the possible bias may not sufficiently confound our results and conclusions because of no significant baseline differences between the CSC and control groups.

In conclusion, our study may provide further evidence for the congestion of vortex vein in the pathogenesis of CSC. UWF SS-OCTA can be used to evaluate the abnormalities of the choroidal structures even at the periphery in eyes with CSC.

## Methods

### Patients

This retrospective observational study was conducted in the Department of Ophthalmology, Peking University People’s Hospital and adhered to the tenets of the Declaration of Helsinki. The protocol was approved by Ethics Committee of People’s Hospital of Peking University.

Patients diagnosed with treatment-naïve unilateral CSC were consecutively included in this study between Jan 2022 and April 2022. Their contralateral eyes were also studied. Age and sex matched healthy subjects were used as controls.

The diagnosis of CSC was based on the presence of subretinal fluid (SRF) and/or PED, with dye leakage from the retinal pigment epithelium on FA and focal choroidal vascular hyperpermeability on the late-phase ICGA. Inclusion criteria of CSC were as follows: (1) presence of SRF involving the fovea with orchoroidal neovascularization (CNV), polypoidal choroidal vasculopathy (PCV), diabetic retinopathy (DR), retinal vein occlusion (RVO), Coats’ disease without PED on OCT, (2) evidence of active leakage on FA, (3) abnormal dilated choroidal vasculature on ICGA, (4) No previous treatments. Participants were excluded if: (1) any other ocular diseases associated with SRF, such as choroidal neovascularization (CNV), polypoidal choroidal vasculopathy (PCV), diabetic retinopathy (DR), retinal vein occlusion (RVO), Coats’ disease and etc.; (2) myopia with the spherical equivalent < -6 diopters, hyperopia > + 3 diopters (spherical equivalent was defined as the sum of the spherical power and half of the cylinder power); (3) any disease that may affect the quality of imaging (quality of OCT or OCTA < 7) such as cataract, high myopia, or nystagmus; (4) severe kidney or liver dysfunction and/ or unstable cardiac disease, (5) pregnancy, (6) any conditions rendering patients intolerable to image acquisitions, (7) or images with severe artifacts preventing accurate analysis.

The aCSC is defined as an acute-onset, dome-shaped serous detachment of the neuroretina, with spontaneous complete resolution of the SRF in 6 months and a good visual prognosis. The cCSC was diagnosed based on the duration of persistent fluid for at least 6 months, as well as multifocal leakage, widespread RPE alterations and photoreceptor alterations^42^.

All included participants underwent a full ophthalmic examination, including measurement of best corrected distance visual acuity (BCVA), intraocular pressure (IOP), axial length (AL), spherical equivalent, slit lamp examination, indirect ophthalmoscopy, UWF SS-OCT and UWF SS-OCTA. BCVA was measured monocularly using a Snellen chart and then converted to Logarithm of the Minimum Angle of Resolution (log MAR) scale for statistical evaluation. AL were obtained from the IOLMaster (Zeiss 700; Carl Zeiss Meditec, Inc, Dublin, CA). Additionally, patients with CSC underwent color fundus photography, FA (Optos 200Tx Optos plc, Dunfermline, United Kingdom) and ICGA (the Spectralis HRA + OCT device, Heidelberg Engineering, Heidelberg, Germany). Data on baseline demographics (sex, age, duration of symptoms, history of hypertension, history of diabetes mellitus, etc.) and ophthalmologic examination findings were collected.

### Evaluation of choroidal parameters by UWF SS-OCTA

Choroidal structures were examined using UWF SS-OCTA (BM-400 K BMizar, TowardPi Medical Technology, Beijing, China), with the combination of long wavelength (1060 nm) full range swept source and 400 kHz A-scan. We obtained 3-dimensional data of vertical 20 mm × horizontal 24 mm × scan depth 6 mm (1536 A-scans at 1280 B-scan positions). Both Bruch’s membrane and choroid-sclera interface were identified automatically with the built-in software. We manually verified the accuracy of automatic segmentation with B-scans if necessary. When measuring the choroidal vasculature, we unified the measurement range among participants by correcting the AL-related magnification using the modified Littmann formula (Bennett procedure)^43,44^.

We set the choroidal thickness as the vertical distance from the Bruch’s membrane (BM) to the chorioscleral interface (CSI). Choroidal volume was defined as the volume from the outer border of the RPE-Bruch’s membrane complex to the choroid-sclera interface which was calculated by the built-in software. The density of the vessel layer was automatically calculated as the ratio of the pixel areas of the vessels divided by the total area of the regions. Large-vessel choroidal layer was the slab between the CSI and 29 µm beneath the BM, and choriocapillaris layer was the slab between the BM and 29 µm beneath the BM.

The measurement position was always centered at the fovea without any rotation, comprising 9 subfields (superotemporal, upper, superonasal, temporal, central, nasal, inferotemporal, lower, inferonasal regions), considering the segmental nature of the choroidal vasculature and vortex veins^44–47^. (Fig. 1).

### Statistical analysis

All statistical analyses were performed with Stata/SE 15.0 (V.15.0; Stata, College Station, TX, USA). For patient characteristics, descriptive methods, with standard summary statistics including the mean (S.D., standard deviation), median, interquartile range (IQR), and proportions were applied. Propensity score matching method was used to generate age and sex matched controls with SPSS (SPSS version 24). Comparisons between healthy subjects and patients with CSC were performed using the unpaired t test for parameters with normal distribution and the Mann–Whitney U test for parameters with nonnormal distribution, as well as the comparisons between aCSC and cCSC. Comparisons between eyes with CSC and fellow eyes were performed using the paired t test for parameters with normal distribution and the Wilcoxon signed rank test for parameters with nonnormal distribution. Correlation analysis was conducted among the choroidal parameters (choriocapillaris density, large-choroidal vessel density, choroidal thickness and choroidal volume). P values of < 0.05 were considered statistically significant.

### Ethical approval

All procedures performed in studies involving human participants were in accordance with the ethical standards of the Institutional Review Board of the Peking University People’s Hospital and with the 1964 Helsinki declaration and its later amendments or comparable ethical standards.

### Informed consent

Informed consent was obtained from all individual participants included in the study.

## Supplementary Information

Supplementary Information.

## Data Availability

Data are available upon reasonable request. Deidentified participant data that underline the results reported in this article (text, tables and figures) could be shared upon reasonable request sent to the corresponding author.
