# Supplementary material for: Quantitative analysis of choroidal vasculature in central serous chorioretinopathy using ultra-widefield swept-source optical coherence tomography angiography
Source: Sci Rep. 2022 Nov 1;12:18427. doi: 10.1038/s41598-022-23389-1 (PMC9626581; doi:10.1038/s41598-022-23389-1)
Supplement: Supplementary file 1 — Supplementary Information. [file 41598_2022_23389_MOESM1_ESM.docx]

| Supplementary Table S1 Characteristics of patients with acute CSC and chronic CSC. | | | |
| --- | --- | --- | --- |
| Parameters | Acute CSC | Chronic | P-value |
| No. (male/female) | 34 (25/9) | 15 (11/4) | 0.989 |
| No. of eyes | 34 | 15 | NA |
| Age, years, mean±SD | 43.4±7.5 | 50.3±11.3 | 0.014* |
| Hypertension, n (%) | 4 (11.8) | 2 (13.3) | 0.877 |
| Diabetes, n (%) | 1 (2.9) | 0 (0) | 0.502 |
| Duration of symptoms, months, median (IQR) | 2 (1, 3) | 8 (6, 12) | <0.001* |

| Supplementary Table S2 Comparisons of parameters between acute and chronic CSC eyes. | | | | |
| --- | --- | --- | --- | --- |
|  | acute eyes | chronic eyes | P-value |  |
| Best corrected visual acuity in logMAR, mean±SD | 0.13±0.15 | 0.47±0.53 | 0.003* |  |
| Intraocular pressure, mmHg, mean±SD | 15.0±3.0 | 13.8±2.1 | 0.172 |  |
| Axial length, mm, mean±SD | 23.9±0.8 | 23.6+1.2 | 0.565 |  |
| Spherical equivalent, diopters, median, IQR | 0 (-0.75,0) | 0 (-0.75, 1) | 0.582 |  |
| CSC, central serous chorioretinopathy; logMAR, Logarithm of the Minimum Angle of Resolution; SD, standard deviation  *statistically significant | | | | |

| Supplementary Table S3 Comparisons of choroidal vasculature in nine regions between acute and chronic CSC eyes. | | | | |
| --- | --- | --- | --- | --- |
|  |  | Acute | Chronic | P-value |
| Large choroid vessel density, %, mean±SD | | | |  |
|  | Superotemporal | 72.8±2.1 | 71.3±4.1 | 0.196 |
|  | Upper | 71.3±1.9 | 69.9±2.5 | 0.034* |
|  | Superonasal | 72.9±1.8 | 69.9±4.5 | 0.002* |
|  | Temporal | 71.9±1.5 | 70.1±2.5 | 0.004* |
|  | Central | 72.6±2.0 | 70.7±3.4 | 0.017* |
|  | Nasal | 70.5±2.6 | 67.9±7.3 | 0.354 |
|  | Inferotemporal | 72.0±2.5 | 69.3±4.0 | 0.006* |
|  | Lower | 70.8±2.3 | 69.1±3.8 | 0.302 |
|  | Inferonasal | 70.5±3.0 | 67.5±6.9 | 0.345 |
|  | Average | 71.7±1.7 | 69.5±3.9 | 0.155 |
| Choroidal thickness, um, mean±SD | | |  |  |
|  | Superotemporal | 287.0±65.7 | 261.5±62.1 | 0.208 |
|  | Upper | 331.3±89.0 | 274.7±76.4 | 0.048* |
|  | Superonasal | 284.8±92.5 | 223.8±76.6 | 0.013* |
|  | Temporal | 275.3±55.4 | 221±60.7 | 0.004* |
|  | Central | 369.4±97.4 | 307.3±119.4 | 0.061 |
|  | Nasal | 249.1±83.6 | 218.4±93.0 | 0.257 |
|  | Inferotemporal | 240.4±59.4 | 208.2±55.6 | 0.082 |
|  | Lower | 239.7±56.6 | 205.7±73.7 | 0.084 |
|  | Inferonasal | 171.6±41.7 | 155.8±49.3 | 0.252 |
| Choroidal volume, mm3, mean±SD | | |  |  |
|  | Superotemporal | 12.5±2.9 | 11.4±2.7 | 0.202 |
|  | Upper | 17.7±4.8 | 14.7±4.1 | 0.046* |
|  | Superonasal | 12.4±4.0 | 9.7±3.4 | 0.014* |
|  | Temporal | 14.7±3.0 | 11.8±3.2 | 0.004* |
|  | Central | 19.7±5.2 | 16.4±6.4 | 0.06 |
|  | Nasal | 13.3±4.5 | 11.7±5.0 | 0.257 |
|  | Inferotemporal | 10.9±2.7 | 9.5±2.6 | 0.083 |
|  | Lower | 12.8±3.0 | 11.0±3.9 | 0.084 |
|  | Inferonasal | 7.8±1.9 | 7.1±2.3 | 0.256 |
| Choriocapillaris density, %, mean±SD | | |  |  |
|  | Superotemporal | 46.9±1.9 | 47.6±2.0 | 0.0677 |
|  | Upper | 47.2±1.2 | 47.2±1.1 | 0.05 |
|  | Superonasal | 48.2±1.4 | 47.7±1.7 | 0.201 |
|  | Temporal | 45.8±1.5 | 46.3±1.3 | 0.254 |
|  | Central | 46.0±1.2 | 45.9±1.1 | 0.652 |
|  | Nasal | 47.1±1.3 | 46.9±1.2 | 0.459 |
|  | Inferotemporal | 45.7±1.9 | 47±1.7 | 0.03* |
|  | Lower | 47.1±1.5 | 47.5±1.4 | 0.218 |
|  | Inferonasal | 47.3±1.4 | 47.1±2.4 | 0.724 |
|  | Average | 46.8±1.1 | 47.0±1.1 | 0.55 |

| Data are presented as means±standard deviations unless otherwise indicated. |
| --- |

Supplementary Table S4 Comparisons of choriocapillaris density between central and extra-central subfields in acute and chronic CSC eyes.

| Variable | Groups | Extra-central | Central | P-value |
| --- | --- | --- | --- | --- |
| Choriocapillaris density, % | acute, mean±SD | 46.9±1.1 | 46.0±1.2 | <0.001* |
|  | chronic, mean±SD | 47.2±1.2 | 45.9±1.1 | 0.002* |

CSC, central serous chorioretinopathy

Data are presented as means±standard deviations unless otherwise indicated.
